# Rasch Analysis of Authentic Evaluation of Young Children's Functioning in Classroom Routines

**DOI:** 10.3389/fpsyg.2021.615489

**Published:** 2021-03-29

**Authors:** Catalina Patricia Morales-Murillo, Pau García-Grau, R. A. McWilliam, Ma Dolores Grau Sevilla

**Affiliations:** ^1^Universidad Internacional de La Rioja, Logroño, Spain; ^2^Campus Capacitas-Catholic University of Valencia San Vicente Mártir (UCV), Valencia, Spain; ^3^Catholic University of Valencia San Vicente Mártir, Valencia, Spain; ^4^University of Alabama, Tuscaloosa, AL, United States

**Keywords:** Rasch analysis, reliability, validity, authentic assessment, preschool, child functioning

## Abstract

This study evaluated the functioning of children in early childhood education classroom routines, using the 3M Functioning in Preschool Routines Scale. A total of 366 children aged 36 to 70 months and 22 teachers from six early childhood education centers in Spain participated in the study. The authors used the Rasch model to determine the item fit and the difficulty of the items in relation to children's ability levels in this age range. The Rasch Differential Item Functioning (DIF) analysis by child age groups showed that the item difficulty differed according to the children's age and according to their levels of competence. The results of this study supported the reliability and validity of the 3M scale for assessing children's functioning in preschool classroom routines. A few items, however, were identified as needing to be reworded and more difficult items needed to be added to increase the scale difficulty level to match the performance of children with higher ability levels. The authors introduced the new and reworded items based on the results of this study and the corresponding ICF codes per item. Moreover, the authors indicate how to use the ICF Performance Qualifiers in relation to the 3M scale response categories for developing a functioning profile for the child.

Researchers in the early childhood education and the early intervention fields have noted that conventional testing misrepresents children's true abilities (Neisworth and Bagnato, 2004; Bagnato et al., 2010). Authentic assessment represents an alternative to traditional testing, for capturing children's true functioning skills (Bagnato et al., 2010).

Authentic evaluation has been defined as the “systematic record of developmental observation over time by families and knowledgeable caregivers about the naturally occurring competencies of young children in daily routines” (Bagnato and Yeh-Ho, 2006, p. 67). It differs from conventional evaluation because it takes place in the child's natural environment, where a child's caregiver observes the child's responses to the demands of daily routines (Bagnato, 2007). This type of evaluation focuses on the functioning of the child in natural contexts rather than assessing isolated skills by unfamiliar people in unfamiliar places (Bronfenbrenner, 1976; Meisels et al., 2001; Bagnato, 2005).

The experiences of children in natural environments interact with their biological dispositions to promote their competence (Shonkoff and Philips, 2000; Shonkoff, 2010; Center on the Developing Child at Harvard University, 2017). Therefore, children learn and develop as a result of their functioning in daily activities at home, school, and community (McWilliam, 2016). The contingent responses children receive from the adults in their natural environments strengthen their mastery, sense of competence, task orientation, participation, learning, and development (MacTurk and Morgan, 1995). As García-Grau (2015) indicated, the objectives of the intervention, in natural contexts, should be aimed at trying to increase, as far as possible, the number and frequency of opportunities for participation and functioning of the child (Dunst et al., 2001). Children show greater competence and engagement in learning contexts where exploration and successful participation of children in daily activities are encouraged and supported (Wachs, 1979, 2000; Kontos et al., 2002; Booren et al., 2012; Fuligni et al., 2012; Veiga et al., 2012; Vitiello and Williford, 2016).

Experts have recommended using authentic evaluation to plan interventions to promote children's functioning, development, and learning in daily routines (Bagnato et al., 2010). An authentic evaluation has greater social validity compared to conventional developmental tests (Bagnato et al., 2014). It is adjusted to the developmental level of each child, the functioning capabilities of the child, the demands of the routines, and the report of the adults in the environment (Bronfenbrenner and Morris, 1998; Bagnato and Neisworth, 2000). Moreover, teachers' assessment of children's competence when participating in classroom activities can be reliable (Meisels et al., 2001).

We identified *functioning* as a key aspect of authentic evaluation and as a key step toward learning. Intervention aimed at functioning (i.e., engagement) makes learning possible (McWilliam et al., 1985). Functioning can be regarded as the participation of children in the activities they encounter daily (Granlund, 2013; Coelho and Pinto, 2018). As pointed by Coelho and Pinto (2018), the International Classification of Functioning, Disability, and Health (ICF; World Health Organization, 2001) is regarded as a useful tool for determining children's functioning and participation in routines. The ICF perspective discourages the use of norm-reference tools to assess children's functioning and recommends the use of contextualized and comprehensive assessments that focus on the interactions of the child with the environment (i.e., adults/peers/materials).

From the International Classification of Functioning, Disability, and Health (ICF; World Health Organization, 2001), the child capacities and performance are considered. Child capacities are determined by what the child can do taking into consideration his or her body structures and functions. When considering the degree to which the child is able to participate in routines when offered supports from the environment, then child performance can be determine.

Following the ICF perspective, Boavida and McWilliam (2015) stated that for a child to function in a context, there must be a match between (a) the child's abilities, which correspond to the child's competence (what is the child capable to do); (b) the demands of the routines (represent the facilitators or barriers to the child's participation), which prevents or facilitates the participation of the child; (c) the interests of the child, which refers to how interesting the activity is to the individual child; and (d) the functional domains, which encompass the appropriate participation of the child in the routines (activities and participation). By considering these components, we can determine how a child functions in the classroom, we can determine the child's level of participation in the different routines and then evaluate which the barriers or facilitators for the child's meaningful participation in the routine. Therefore, we can plan interventions or supports aimed at increasing the child's participation in the classroom routines (see Figure 1). These interventions are aimed at promoting child performance and determining the difficulty level for a child to functioning in natural environments (i.e., home, community, and school).

**Figure 1:**
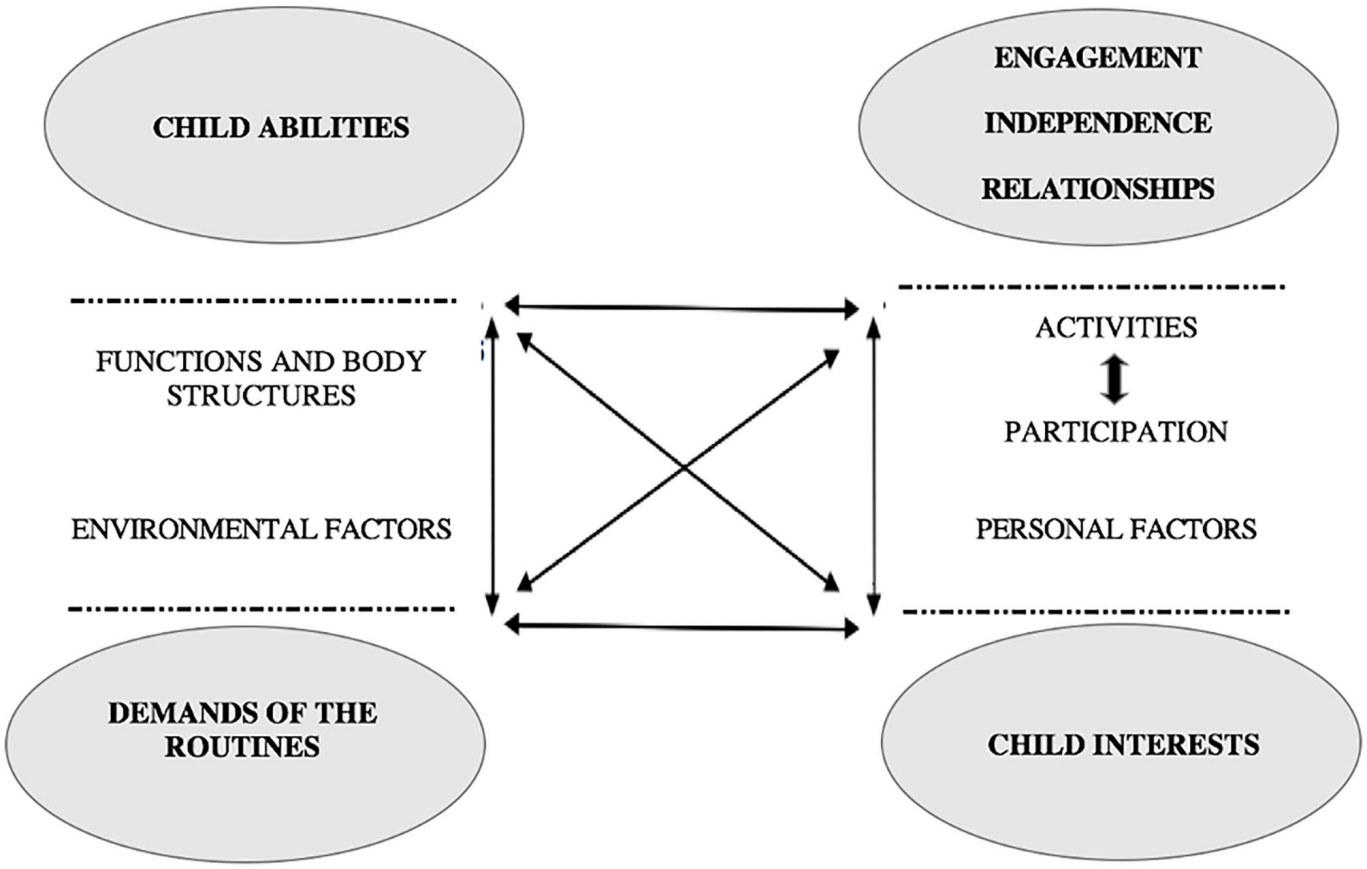
Components of child functioning from an ICF and MEISR perspective (McWilliam and Younggren, 2019).

Among the tools to assess children's functioning in the preschool classroom, we identified the Measurement of Engagement, Independence, and Social Relations (MEISR; McWilliam and Younggren, 2019), the 3M Functioning in Preschool Routines Scale (Morales Murillo and McWilliam, 2014), the Classroom Measurement of Engagement, Independence and Social Relations (ClaMEISR; McWilliam, 2014), and the Matrix for Assessment of Activities and Participation (Castro and Pinto, 2015).

These instruments are consistent with the premise of *functioning* in the International Classification of Functioning, Disability, and Health (ICF, World Health Organization, 2001) each item relates to child performance, the routines represent the context where the activity or participation is supposed to happen. Through the ratings, we can identify the participation of the child in the routine, and then being able to determine if there are barriers or facilitators to participation of the child. The first three instruments include, in addition, the functional domains of development (i.e., engagement, independence and social relationships) (McWilliam, 2010). Moreover, these three tools allow for the assessment of children's competencies in the contexts (i.e., routines) where the abilities are necessary. Therefore, teachers can provide the supports children need in those routines. The 3M represents a short version of the ClaMEISR and evaluates the functioning of children in early childhood education classroom routines.

A previous study identified the psychometric properties, using factor analysis, of the 3M for Spanish participants (Morales Murillo et al., 2018). Besides the factor structure and psychometric characteristics, however, item-level analysis (Rasch, 1960, 1980) can provide more detailed scale information (Snyder and Sheehan, 1992; Chien and Bond, 2009; DiStefano et al., 2014). It would allow us to test the construct validity of the tool and to identify the adequacy of the items to the ability level of children aged 3–5 years. Moreover, the Rasch model complements traditional factor analysis (Linacre, 2005) in providing category-related information (García-Grau et al., 2021). We can determine if the four categories of the 3M fit the scores of children's competencies in this age range. Adjusting both the scale items (i.e., by deleting, adding or reformulating items based on the Rasch results) and the scale of measurement (i.e., by deleting or adding one more level to the measurement scale) improves the scale for validation in a large population. Whereas item-fit to the Rasch model, item-person maps, and the analysis of the scale categories (response options) are commonly reported elements in other studies with authentic assessment tools and early childhood development evaluations (Elbaum et al., 2010; Curtin et al., 2016; Nasir-Masran et al., 2017), these have not received much attention with tools assessing child functioning in routines in early childhood (i.e., ClaMEISR, MEISR, 3M scale).

Rasch is one of the most widely used and accepted model from the set of probabilistic item response theory (IRT) models (Muñiz, 2010). The Rasch analysis software uses a logarithmic transformation of the item and person data to convert the ordinal data scores into interval data (Bond and Fox, 2007). It assumes invariant, unidimensional linear measurement; in other words, it does not assume that all the items are equally difficult. Thus, a *correct* response is a logistic mathematical function between respondent's trait level and the difficulty of the items (Muñiz, 1989; Zucca et al., 2012). This analysis provides information about the difficulty of the items, the relative fit, information about the individual measurement errors and biased and inappropriate items (Fitzpatrick et al., 2003; Tennant and Conaghan, 2007; Vélez et al., 2016).

The partial-credit model is used in Rasch analysis to compare persons and items and is applicable for Likert-type scales, because it allows different thresholds for different items (Masters, 1982; Vélez et al., 2016). For the 3M, a rating of 1 indicates the child “not yet” performs the skill, 2 indicates the child “rarely” performs the skill, 3 indicates the child performs the skill “often,” and 4 indicates the child performs the skill “almost all the time.” The Rasch model provides a scaled interval-based map of persons and items, which allows the researcher visually to relate the difficulty of the item (placed on the right side of the map, with the most difficult items at the top) and the person's ability (on the left side, with respondents with the highest ability at the top of the map).

We identified this analytical method as the next step to improve the 3M scale items. We looked at the reliability, item fit, and adequacy of response categories. In addition, we analyzed the sensitivity of the scale items to differentiate children's abilities at 3, 4, and 5 years of age. Based on these results, we adjusted the scale items, added its corresponding ICF codes and cross-walked the 3M response categories with the ICF Performance Qualifiers to be able to produce a child functioning profile.

## Methods

### Participants

A total of 366 children attending either public (*n* = 162) or private (*n* = 204) early childhood education centers in Spain participated in the study. Three hundred and twenty-three (89%) of the 366 children were born in Spain, and 32 (9%) were born in other countries, such as Romania, Portugal, and Colombia. For nine children (2%), no nationality was reported.

The children's ages ranged from 36 to 70 months (*M* = 52.70, *SD* = 9.34). Teachers reported 12 children had a diagnosed disability, including autism and language and developmental delays. Most children came from families with a middle income (*n* = 294), followed by those with a lower income (*n* = 43), and those with a higher income (*n* = 27). Teachers reported that 257 of the 366 children lived with both parents, and 70 children lived with divorced parents, foster parents, or one parent had died. Twelve children lived with single mothers, and two lived with a single father. Data on the legal guardian or caregiver were missing for 22 children.

### Instrument

We used the 3M Functioning in Preschool Routines Scale (Morales Murillo and McWilliam, 2014) for this study. The 3M asks teachers to rate 25 items on a 4-point rating scale from 1 = *not yet* to 4 = *almost all the time*. The 25 items are structured in 5 common preschool classroom routines: meal time, free play, toileting, art, and teacher-led activities, with 5 items per routine. With exploratory factor analysis, we have identified four underlying factors: Self-Help, Average Engagement, Personal-Social, and Sophisticated Engagement (Morales Murillo et al., 2018).

The 3M produces 10 different child functioning scores in preschool classroom routines: a 3M total score, four factor scores, and five preschool classroom routine-scores). Each of the scores represents a mean of all item scores. In addition, the percentage of mastered items (those items scored as 4) can be calculated to reflect the child's mastery of skills for the total scale, for each factor, and for each routine. Items scored as 2s or 3s might reflect skills in the zone of proximal development and could be considered for scaffolding (Vygotsky, 1993). The scale is not intended to be a curriculum-based assessment nor is it an exhaustive repertoire of all possible functional skills of children in early childhood education programs. The 3M is meant to produce a functioning profile for children that could guide teachers to consider those routines where the children might need supports.

Morales Murillo et al. (2018) found high internal consistency for the 3M total score (α = 0.96) and the scores of its factors. The Cronbach α values for the factor scores were 0.81, 0.86, 0.92, and 0.95 for Self-Help, Average Engagement, Personal-Social, and Sophisticated Engagement, respectively.

### Procedures

First, we obtained institutional review board (IRB) approval. Next, we contacted the principals of the early childhood education (ECE) centers via email and later through a meeting to provide information on the objectives and the procedure of the investigation. This was a convenience sample and the centers were contacted through collaborators of our university. After the principals confirmed their ECE center participation, we met with the teachers of the 3-, 4-, and 5-year-old classrooms to explain the objectives and procedure of the project and to provide them with informed-consent forms for them and the children's legal guardians to sign. Once the legal guardians provided consent forms, the teachers completed a 3M scale for each child in their classroom. When the 3M scales were completed, the principals contacted the researchers to collect the signed consent forms and the completed scales.

### Data Analysis

We entered and organized the data using the Statistical Package for Social Sciences SPSS v24 and analyzed descriptive statistics. For the Rasch analysis, we used WINSTEPS (Linacre, 2021) to perform the partial-credit model (Masters, 1982), the reliability and separation of respondents and items (internal consistency), and the maps and thresholds analyses.

The item fit to the Rasch model was assessed through the mean square residuals (MNSQ). The infit and outfit mean square statistics indicate unexpected answers near and far from the respondent's measure level (i.e., person logit), respectively. They provide evidence of construct validity (Linacre, 2006).

Finally, the potentially biased items according to the age of children (36–48, 49–60, and 60–72 months) were analyzed through DIF analysis using the Rasch–Welch Method (*t*-test), the significance criterion of >0.5 logit difference between groups was used (Belvedere and de Morton, 2010). In addition to avoid Type I errors, it was applied a Bonferroni correction (Linacre, 2013). After the Bonferroni correction for multiple comparisons, we accepted *p* < 0.0007, after dividing our alpha by 69 (number of *t-*tests in the multiple-comparisons analysis). The unidimensionality of the 3M scale was evaluated using principal components analysis of residuals, accepting an explained variance of >50% (Streiner et al., 2015) and unexplained variance <3 eigenvalues for a secondary dimension after the first contrast (Linacre, 2006).

## Results

Ratings on the 3M instrument indicated an overall mean score of 3.39 (*SD* = 0.61) on a 4-point scale. Scores by age group suggest and increasing pattern of the overall score of the scale as age was higher (Table 1).

**Table 1:** 3M mean scores of children.

	**All children (*****N*** **=** **366)**		**3-year-olds (*****n*** **=** **96)**		**4-year-olds (*****n*** **=** **158)**		**5-year-olds (*****n*** **=** **112)**	
	**Min–Max^a^**	***M* (*SD*)**	**Min–Max^a^**	***M* (*SD*)**	**Min–Max^a^**	***M* (*SD*)**	**Min–Max^a^**	***M* (*SD*)**
3M total score	1–4	3.39 (0.61)	1–4	2.9 (0.69)	1–4	3.49 (0.45)	1–4	3.67 (0.49)

a *Lowest and highest score*.

The internal consistency of the 25 original scale items was assessed through the reliability and separation indices of both, items and persons (Belvedere and de Morton, 2010). For the whole scale, the reliability of the items was 0.99 and the separation was 8.51 (item *SE* = 0.19). The reliability for persons was 0.91 and the separation 3.21, (person *SE* = 0.10), and the reliability of the items was >0.70 (KR-20 or Cronbach's Alpha) and separation >2, indicating adequate internal consistency (Ashley et al., 2013; Smith et al., 2013).

The unidimensionality of the 3M scale was analyzed through the Rasch principal-component analysis of residuals, and 84% of the variance in scores was explained by the measure. Because more than three eigenvalues (i.e., factors) were identified in the first contrast (eigenvalue = 3.1), unidimensionality could not be claimed, indicating the existence of more than one dimension in the scale. When considering the KR-20 and separation indices of the factors by persons, results suggested low reliability of the dimensions previously identified using Classical Measurement Theory (Table 2). Because the factors presented low reliability when considering the KR-20 and separation indices for person, and the eigenvalue of the first contrast was close to indicate unidimensionality of the scale (3.1). We proceed to evaluate the fit of the items to the Rasch model to determine if items need to be deleted. This was done in order to rerun the Rasch principal-component analysis of residuals to evaluate the scale unidimentionality with the new set of items.

**Table 2:** Results of the principal-component analysis of residuals.

				**Item**			**Person**		
**Factor**	**Items**	**Explained variance by measure**	**Eigenvalue first Contrast**	**KR-20**	**Separation**	**SE**	**KR-20**	**Separation**	**SE**
Sophisticated engagement	1.3, 1.5, 2.1, 2.2, 2.3, 2.4, 4.4, 5.2	81%	1.9	0.97	6.02	0.32	0.86	2.50	0.15
Self-help	1.1, 1.2, 1.4, 3.1, 3.2, 3.5	90%	1.8	0.99	9.55	0.75	0.56	1.14	0.11
Average engagement	4.5, 5.1, 5.4, 5.5	53%	1.7	0.94	3.82	0.29	0.60	1.20	0.11
Personal–Social	2.5, 3.3, 3.4, 4.1, 4.2, 4.3, 5.3	75%	2.0	0.97	5.33	0.22	0.78	1.88	0.09

The fit of the data to the Rasch model was calculated through the unstandardized information-weighted mean square statistic (infit MNSQ), unstandardized outlier-sensitive mean square statistic (outfit MNSQ), standard error estimates, and point-measure correlations (see Table 3). Only item 3.2 showed a misfit over 1.4 in both infit and outfit MNSQs. Item 3.5 showed an infit MNSQ misfit over 1.4. Item 1.2 was a misfit with an outfit MNSQ value above 1.4. Outfit MNSQ misfit under 0.7 was observed in 5 items (2.1, 2.2, 2.3, 4.4, and 5.2). Considering the infit MNSQ, only 4 items (2.1, 2.3, 4.4, and 5.2) had mean squares below 0.7. When using a less conservative range to determine misfit, 0.5–1.5 range (Linacre, 1999, 2006), item 3.2 did not fit with infit and outfit indices above 1.5, and item 1.2 with an outfit above 1.5. The fact that only two items had a 0.5–1.5 misfit (Handley et al., 2008; Linacre, 2013) provides evidence of the construct validity of the scale (Linacre, 2006).

**Table 3:** First Rasch model item difficulty and fit analysis.

**Item**	***N***	**Item**	**Model**	**Infit**	**Outfit**	**PMC**
		**Difficulty**	**SE**	**MNSQ**	**MNSQ**	
**Meal time**						
1.1. Uses fork and spoon to stab and scoop food	244	−1.21	0.15	0.95	1.07	0.56
1.2. Drinks from cup without spilling content	258	−2.03	0.18	1.02	**1.81**	0.47
1.3. Initiates communication with peers	286	0.03	0.10	0.82	0.81	0.70
1.4. Clears table after eating (throws away trash/puts away food containers) without been prompted	227	1.20	0.10	1.15	1.25	0.67
1.5. Uses words, signs, and/or gestures to express needs to the teacher and peers	286	−0.88	0.13	1.11	0.74	0.59
**Free play**						
2.1. Engages in pretend play by acting out scenarios	332	−0.03	0.09	0.62	0.62	0.72
2.2. Independently chooses and obtains accessible materials	331	−0.21	0.10	0.82	0.62	0.69
2.3. Cooperates with peers while playing (e.g., negotiates roles)	332	0.16	0.09	0.67	0.58	0.74
2.4. Talks to peers using understandable language	332	0.10	0.09	0.96	0.94	0.68
2.5. Shows empathy toward other people's feelings	331	0.92	0.08	1.17	1.21	0.67
**Toileting**						
3.1. Urinates in potty with no accidents	331	−1.85	0.15	1.23	0.97	0.48
3.2. Washes his/her hands after using the potty	331	0.77	0.08	**2.06**	**2.97**	0.40
3.3. Uses zipper, snap, or buttons	329	0.62	0.08	0.93	1.01	0.70
3.4. Dresses and undresses without assistance	325	0.71	0.08	0.94	0.94	0.70
3.5. Goes into bathroom independently or asks for permission by using words or signs	330	−1.81	0.15	**1.48**	0.71	0.48
**Art**						
4.1. Responds to 3-step instructions from the teacher	330	0.44	0.09	1.12	0.96	0.68
4.2. Makes representational art (draws, paints, or builds things to look like real objects)	330	0.93	0.08	0.9	0.76	0.76
4.3. Uses scissors independently	331	1.12	0.08	1.15	1.12	0.72
4.4. Talks about his or her art product in full sentences	330	0.78	0.08	0.65	0.57	0.78
4.5. Waits for his or her turn to use materials without getting upset	330	0.37	0.09	1.32	1.32	0.61
**Teacher-led activities**						
5.1. Attends to teacher when he or she is talking to the group for periods of time longer than 5 min	331	0.16	0.09	1.24	1.2	0.60
5.2. Participates in group activities that involve communication, by using full sentences	331	0.56	0.08	0.66	0.57	0.77
5.3. Jumps by lifting both feet from the ground	331	−0.34	0.10	0.98	0.93	0.64
5.4. Imitates teacher's gestures and movements while singing songs	331	−0.40	0.10	0.88	0.81	0.65
5.5. Follows rules and teacher's requests	331	−0.13	0.10	0.9	0.89	0.65
Model Mean	316.4	0	0.102	1.03	1.01	
Model SD	30	0.908	0.027	0.3	0.49	

*Values in boldface are those considered as a misfit in relationship to the 0.7–1.4 range (Handley et al., 2008; Linacre, 2013). Underscored values represent those items that are not consider as misfit according to the 0.5–1.5 range (Linacre, 1999, 2006)*.

Most of the items showed reliable scores with standard error estimates between 0.08 and 0.18. Six items presented point-measure correlations about 0.70. Experts recommend these range between 0.30 and 0.70 (Allen and Yen, 1979). Therefore, 19 items showed a good discrimination level (Buz and Prieto, 2013). The item difficulty ranged from 1.20 [item 1.4: “Clears table after eating (throws away trash/ puts away food containers”)] to −2.03 (item 1.2: “Drinks from cup without spilling content”; see Table 3).” The higher the value, the more “difficult” the item was and the lower the score it received. Most items (i.e., 21 items) were from below −1 logits to above +1 logits, which makes it difficult to identify children performing at the extreme ends of the log odds unit scale—a desirable characteristic of a scale.

After evaluating the items fit through MNSQ values, it was decided to delete the items which showed a misfit in the MNSQ infit and outfit below 0.50 and above 1.50 (Handley et al., 2008; Linacre, 2013). Therefore, items 3.2 and 1.2 were deleted and the Rasch principal-component analysis of residuals was rerun. The results suggested unidimentionality of the scale items, with an eigenvalue of 2.9 of unexplained variance after the first contract and more than 50% of variance explained by measures (83%).

The items fit to the Rasch model was, again, tested. The results suggested item 3.5 was an infit MNSQ misfit above 1.50. As for the outfit MNSQ values, items 1.4, and 1.1 were a misfit above 1.50. When using the most conservative range 0.70 and 1.40, items 4.2 and 3.5 were a infit misfit above 1.40 and items 4.4, 5.2, 2.3, and 2.1 were a misfit below 0.70. When interpreting the outfit MNSQ results using this more conservative range, items 1.4, 4.5, and 1.1 were a misfit above 1.40. As for outfit MNSQ values below 0.70, items 4.4, 2.3, 2.1, and 2.2 were a misfit. Model error estimates were between 0.08 and 0.16. Point-measure correlations after deleting items 3.2 and 1.2 oscillated between 0.47 and 0.79, with 16 items showing a good discrimination levels with point-measure correlations between 0.30 and 0.70. Item 1.4 continued to be the most difficult item (measure logit = 1.26) and item 3.1 resulted the easiest item (measure logit = −2.01; Table 4).

**Table 4:** Second Rasch model item difficulty and fit analysis after deleting items 3.2 and 1.2 from the original scale.

**Item**	***N***	**Item**	**Model**	**Infit**	**Outfit**	**PMC**
		**difficulty**	**SE**	**MNSQ**	**MNSQ**	
**Meal time**						
1.1. Uses fork and spoon to stab and scoop food	229	−1.32	0.16	1.03	1.51	0.56
1.3. Initiates communication with peers	271	−0.02	0.11	0.85	0.85	0.71
1.4. Clears table after eating (throws away trash/ puts away food containers) without been prompted	212	1.26	0.10	1.32	1.61	0.67
1.5. Uses words, signs, and/or gestures to express needs to the teacher and peers	271	−0.97	0.13	1.17	0.79	0.60
**Free play**						
2.1. Engages in pretend play by acting out scenarios	317	−0.10	0.10	**0.62**	**0.64**	0.73
2.2. Independently chooses and obtains accessible materials	316	−0.28	0.10	0.82	**0.63**	0.70
2.3. Cooperates with peers while playing (e.g., negotiates roles)	317	0.10	0.10	**0.68**	**0.62**	0.74
2.4. Talks to peers using understandable language	317	0.04	0.09	0.99	1.07	0.69
2.5. Shows empathy toward other people's feelings	316	0.92	0.08	1.26	1.35	0.68
**Toileting**						
3.1. Urinates in potty with no accidents	316	−2.01	0.16	1.35	1.35	0.47
3.3. Uses zipper, snap, or buttons	314	0.59	0.09	1.00	1.24	0.70
3.4. Dresses and undresses without assistance	310	0.68	0.09	1.03	1.10	0.70
3.5. Goes into bathroom independently or asks for permission by using words or signs	315	−1.96	0.15	1.58	0.77	0.48
**Art**						
4.1. Responds to 3-step instructions from the teacher	315	0.41	0.09	1.15	0.99	0.69
4.2. Makes representational art (draws, paints, or builds things to look like real objects)	315	0.92	0.08	0.92	0.80	0.77
4.3. Uses scissors independently	316	1.13	0.08	1.21	1.26	0.73
4.4. Talks about his or her art product in full sentences	315	0.76	0.09	**0.67**	**0.62**	0.79
4.5. Waits for his or her turn to use materials without getting upset	315	0.32	0.09	**1.42**	**1.49**	0.61
**Teacher-led activities**						
5.1. Attends to teacher when he or she is talking to the group for periods of time longer than 5 min	316	0.11	0.09	1.33	1.32	0.61
5.2. Participates in group activities that involve communication, by using full sentences	316	0.53	0.09	0.70	**0.61**	0.77
5.3. Jumps by lifting both feet from the ground	316	−0.42	0.10	1.00	0.98	0.64
5.4. Imitates teacher's gestures and movements while singing songs	316	−0.49	0.10	0.91	0.84	0.66
5.5. Follows rules and teacher's requests	316	−0.21	0.10	0.95	0.94	0.66
Model Mean	316.4	0	0.102	1.03	1.01	
Model SD	30	0.908	0.027	0.3	0.49	

*Values in boldface are those considered as a misfit in relationship to the 0.7–1.4 range (Handley et al., 2008; Linacre, 2013). Underscored values represent those items that are consider as misfit according to the 0.5–1.5 range (Linacre, 1999, 2006)*.

For those 23 items, the item KR-20 results suggested strong internal αconsistency of the items (*KR-20* = 0.98; separation = 7.67; *SE* =0.19). At the persons level, internal consistency results suggested strong internal consistency as well (*KR-20* = 0.90; separation = 2.95; *SE* = 0.09).

**Figure 2:**
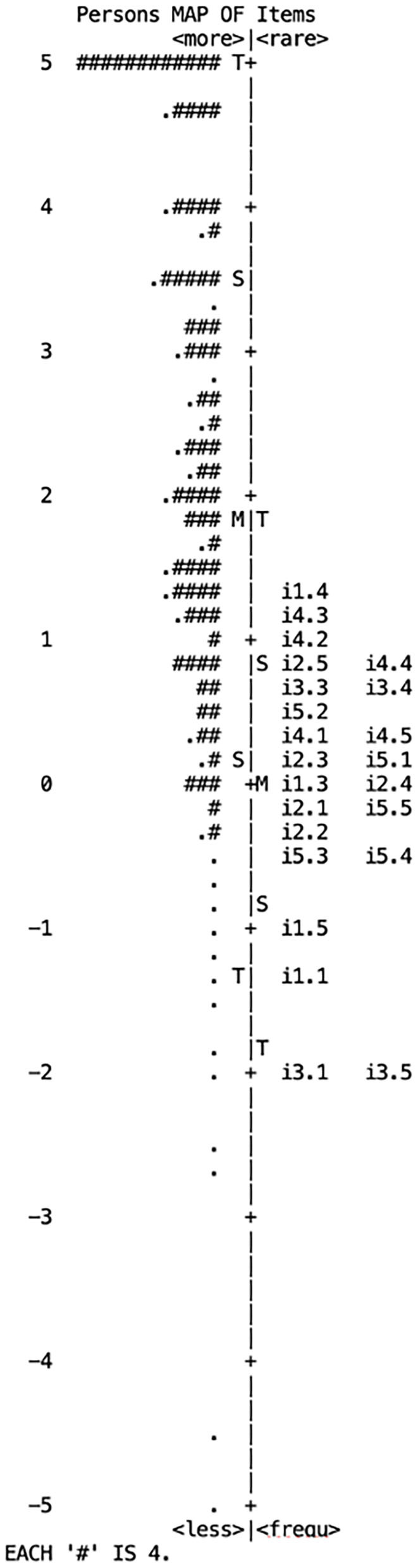
Person-item map of the 3M scale.

When we analyzed persons and items in the same scale in the person-item map (Figure 2), persons' ability level on the left side, represented by #, are related to the item difficulty on the right side. The children's ability level (more ability at the top of the left side of the map) was higher than the average item difficulty (the most difficult items at the top of the right side), indicating that the majority of children mastered the skills described in the 3M items (i.e., items were achieved for that age). Near a 2-logit difference was found between both *M*s, indicating the mean of the respondents' ability level was above the mean of the difficulty level of the items. All items matched the competence level of children. The 3M items were located in a span of 4 logit units, from −2-logit to +2-logit. Items 1.4, 4.3, and 4.2 were the most difficult items above S (1 *SD* from the mean of item difficulty). Items 3.1, 3.5, 1.1, and 1.5 were the easiest items, with all of them 1 *SD* below the mean.

**Figure 3:**
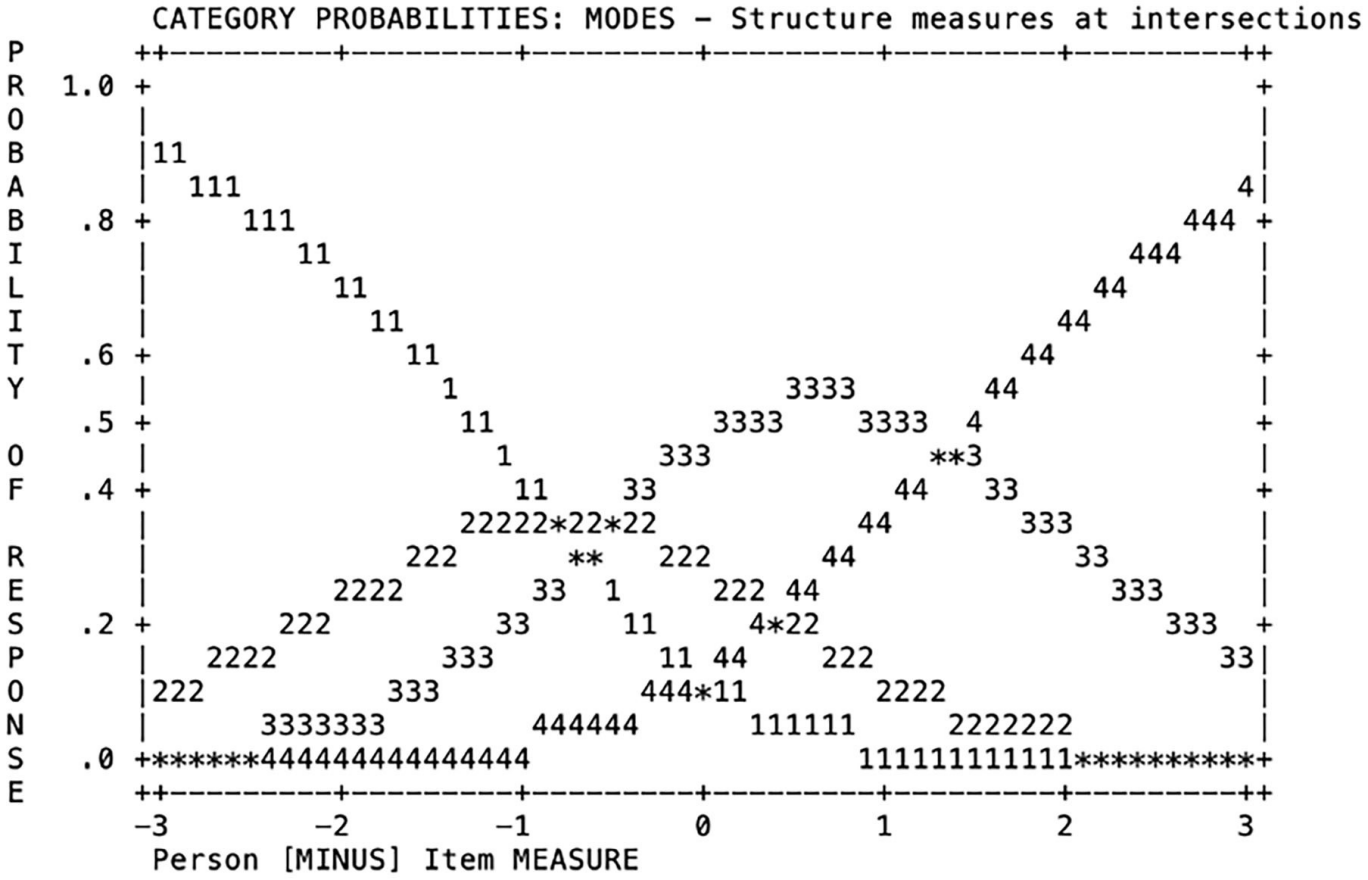
Category (scale scores) probability curves of the 3M scale.

Probability curves were analyzed to study the adequacy of the four categories of response (points on the scale) to the recommended pattern. Figure 3 represents the item thresholds calibration. The Rasch–Andrich threshold parameters were ordered from −0.84, −0.51, to 1.35 (*SE* =0.06, 0.04, and 0.03, respectively). Therefore, the average measures and threshold estimates increased in parallel with the increment across category labels (Arias González et al., 2015). All categories were likely to be chosen, meaning that each category matched a certain ability level of children. Teachers were less likely to choose the 2nd category of response (i.e., Point 2 on the scale). Standard errors of the Rasch–Andrich threshold measure were low, ranging from 0.03 to 0.06, supporting the precision of the estimates (Arias González et al., 2015).

Finally, the results of the DIF analysis supported the sensitivity of the items to differentiate functioning among age groups. We identified differences in item difficulty by age groups. Table 5 summarizes the results of the *t*-test using the Rasch–Welch Method. Eight items showed statistically significant differences between some of the age group comparisons. For example, for item i1.5 (i.e., Uses words, signs, and/or gestures to express needs to the teacher and peers), the difficulty measure was higher for 3-year-olds than 4-year-olds. Such results could be explained by the age of acquisition of the skills been measured by the item. As it will be easier for 4-year-olds to use their words/signs/gestures to express needs than for 3-year-olds.

**Table 5:** Item difficulty comparison by age groups.

									**Rasch–Welch method**				
**Routine**	**Item**	**Item age in months**	**Age group**	**DIF measure**	**DIF error**	**Age group**	**DIF measure**	**DIF error**	**Joint contrast**	***SE***	***t***	***df***	***p***
Eating	i1.1	36–48	3	−2.20	0.30	4	−0.83	0.20	−1.36	0.36	−3.73	178	**0.0003**
						5	−1.19	0.39	−1.01	0.49	−2.04	144	0.0430
			4	−0.83	0.20	5	−1.19	0.39	0.35	0.44	0.81	218	0.4148
	i1.3	60–72	3	−0.01	0.18	4	0.04	0.15	−0.05	0.23	−0.21	224	0.8330
						5	−0.28	0.30	0.27	0.35	0.77	160	0.4398
			4	0.04	0.15	5	−0.28	0.30	0.32	0.33	0.96	248	0.3401
	i1.4	48–60	3	0.50	0.23	4	1.36	0.13	−0.86	0.26	−3.33	172	0.0011
				0.54	0.22	5	1.70	0.22	−1.20	0.31	−3.82	110	**0.0002**
			4	1.36	0.13	5	1.70	0.22	−0.34	0.25	−1.33	194	0.1837
	i1.5	36–48	3	−1.75	0.23	4	−0.46	0.17	−1.29	0.29	−4.48	224	**0.0000**
						5	−0.91	0.36	−0.83	0.43	−1.96	160	0.0521
			4	−0.46	0.17	5	−0.91	0.36	0.45	0.39	1.15	248	0.2508
Free play	i2.1	42–48	3	−0.08	0.15	4	−0.12	0.15	0.04	0.21	0.19	252	0.8502
						5	−0.17	0.24	0.10	0.28	0.34	206	0.7329
			4	−0.12	0.15	5	−0.17	0.24	0.06	0.28	0.20	268	0.8445
	i2.2	60–71	3	−0.41	0.15	4	−0.16	0.22	−0.25	0.22	−1.14	252	0.2543
						5	−0.28	0.25	−0.13	0.29	−0.46	205	0.6467
			4	−0.16	0.15	5	−0.28	0.25	0.12	0.29	0.40	267	0.6924
	i2.3	48–60	3	0.11	0.14	4	0.08	0.15	0.03	0.21	0.15	252	0.8830
						5	0.09	0.22	0.02	0.27	0.08	206	0.9336
			4	0.08	0.15	5	0.09	0.22	−0.01	0.27	−0.04	268	0.9709
	i2.4	48–60	3	0.09	0.14	4	−0.12	0.15	0.21	0.21	1.00	252	0.3195
						5	0.24	0.22	−0.14	0.26	−0.56	206	0.5779
			4	−0.12	0.15	5	0.24	0.22	−0.35	0.26	−1.34	268	0.1808
	i2.5	36–48	3	0.53	0.14	4	1.35	0.12	−0.82	0.18	−4.47	251	**0.0000**
						5	0.46	0.20	0.07	0.25	0.29	205	0.7755
			4	1.35	0.12	5	0.46	0.20	0.89	0.24	3.78	268	**0.0002**
Toileting	i3.1	60–72	3	−1.96	0.21	4	−2.09	0.28	0.13	0.35	0.37	205	0.7118
						5	−2.02	0.42	0.05	0.47	0.12	205	0.9080
			4	−2.09	0.28	5	−2.02	0.42	−0.07	0.50	−0.15	268	0.8814
	i3.3	48–60	3	0.83	0.14	4	0.56	0.13	0.27	0.19	1.40	249	0.1623
						5	0.09	0.22	0.74	0.26	2.80	204	0.0055
			4	0.56	0.13	5	0.09	0.22	0.47	0.26	1.81	267	0.0710
	i3.4	48–60	3	0.96	0.14	4	0.64	0.13	0.32	0.19	1.64	246	0.1014
						5	0.15	0.22	0.81	0.26	3.07	201	0.0025
			4	0.64	0.13	5	0.15	0.22	0.49	0.26	1.90	265	0.0579
	i3.5	36–42	3	−1.79	0.20	4	−2.26	0.29	0.46	0.36	1.30	250	0.1954
						5	−2.02	0.42	0.23	0.46	0.49	204	0.6275
			4	−2.26	0.29	5	−2.02	0.42	−0.24	0.51	−0.47	268	0.6409
Art	i4.1	48–60	3	0.49	0.14	4	0.08	0.15	0.41	0.20	2.02	250	0.0448
						5	0.84	0.19	−0.35	0.24	−1.50	205	0.1344
			4	0.08	0.15	5	0.84	0.19	−0.77	0.24	−3.20	267	0.0015
	i4.2	48–60	3	1.48	0.14	4	0.46	0.13	1.03	0.20	5.20	250	**0.0000**
						5	0.91	0.19	0.57	0.24	2.40	204	0.0172
			4	0.46	0.13	5	0.91	0.19	−0.46	0.23	−2.00	268	0.0465
	i4.3	48–60	3	2.45	0.16	4	0.40	0.14	2.05	0.21	9.63	251	**0.0000**
						5	0.54	0.20	1.91	0.26	7.36	205	**0.0000**
			4	0.40	0.14	5	0.54	0.20	−0.14	0.24	−0.58	268	0.5632
	i4.4	36–48	3	1.09	0.14	4	0.58	0.13	0.51	0.19	2.66	250	0.0083
						5	0.50	0.20	0.59	0.25	2.39	204	0.0177
			4	0.58	0.13	5	0.50	0.20	0.08	0.24	0.32	268	0.7456
	i4.5	48–60	3	0.01	0.15	4	0.61	0.13	−0.61	0.20	−3.08	250	0.0023
						5	0.24	0.22	−0.23	0.26	−0.89	204	0.3756
			4	0.61	0.13	5	0.24	0.22	0.37	0.25	1.49	268	0.1378
Teacher–led activities	i5.1	36–48	3	−0.66	0.16	4	0.27	0.14	−0.93	0.21	−4.37	251	**0.0000**
						5	1.08	0.18	−1.74	0.24	−7.23	205	**0.0000**
			4	0.27	0.14	5	1.08	0.18	−0.82	0.23	−3.57	268	**0.0004**
	i5.2	48–60	3	0.41	0.14	4	0.60	0.13	−0.19	0.19	−0.97	251	0.3344
						5	0.58	0.20	−0.17	0.25	−0.70	205	0.4829
			4	0.60	0.13	5	0.58	0.20	0.01	0.24	0.06	268	0.9509
	i5.3	36–42	3	−0.14	0.15	4	−0.82	0.18	0.68	0.24	2.85	251	0.0048
						5	−0.42	0.25	0.28	0.30	0.93	205	0.3514
			4	−0.82	0.18	5	−0.42	0.25	−0.40	0.31	−1.27	268	0.2047
	i5.4	36–48	3	−0.71	0.16	4	−0.24	0.16	−0.48	0.23	−2.11	251	0.0359
						5	−0.55	0.26	−0.16	0.31	−0.53	205	0.5989
			4	−0.24	0.16	5	−0.55	0.26	0.31	0.31	1.02	268	0.3107
	i5.5	40–60	3	−0.79	0.16	4	0.19	0.14	−0.98	0.22	−4.52	251	**0.0000**
						5	0.09	0.22	−0.89	0.28	−3.20	205	0.0016
			4	0.19	0.14	5	0.09	0.22	0.09	0.26	0.36	268	0.7227

*Values in bold face are those considered Statistically significant after Bonfenrroni correction (p < 0.0007)*.

## Discussion

This study, using the RASCH model, supported the psychometric properties of the 3M found with traditional factor analyses (Morales Murillo et al., 2018). The internal consistency, in terms of reliability and separation of both persons and items, was acceptable, but we did not find it to be a unidimensional instrument, as defined by the Rasch model, for all 25 items. After deleting two misfitting items (3.2 and 1.2); the results of the unexplained variance after the first contrast (2.9) supported the unidimensionallity of the scale. This dimension is identified as *participation*, which according to the ICF definition of functioning, represents one of the components that is bidirectionally related to the child's body functions and structures, and it is impacted by personal (i.e., interests) and contextual factors (World Health Organization, 2001). Teachers are encouraged to use this scale to determine the levels of participation of the child based on different early childhood classroom routines.

Although the item difficulty span had four complete logit odds units, only five items were not located between −1 and +1 logits. The children's average ability level was 2 *SD* higher than the mean of the item difficulty, meaning that, in general, children achieved the skills described in the 3M, as reported by teachers. Including more difficult items to match the ability of high-level respondents on the left side of the map would help shrink the almost-2-logit difference between both *M*s. In addition, including more difficult items would help raise the “ceiling” of the test (Bond and Fox, 2007, 2015). Nonetheless, we can be confident of the scores for children between −1 logits and +1 logits, because of the number of items in this range.

Because the scale was meant to identify children levels of functioning in classroom routines, the difficulty level matches what children should have mastered between the 3 and 6 years of age (i.e., general average competence levels). Our results indicated that most items in the 3M scale are aimed at a medium level of difficulty, and if we wanted to evaluate more sophisticated forms of competence during classroom routines, items with greater difficulty levels should be added.

We aimed to complement traditional analyses carried out with the 3M scale by giving detailed scale information with Rasch parameters. After a first analysis of items fit through MNSQ values, two items were eliminated from the original scale (items 3.2 and 1.2). Then, in a second analysis of reliability of the items and fit to the Rasch model without items 3.2. and 1.2, results indicated an overall adequate internal consistency of and an adequate fit to the Rasch model, with the exception of eight items (three most difficult and five most easy) when using 0.7 and 1.4 as misfit cut-off points (Handley et al., 2008; Linacre, 2013). When using a more liberal range, 0.5–1.5 (Linacre, 2000, 2006), three items were identified as a misfit (items 3.5, 1.4, and 1.1). These items should be considered for further inspection to ensure no unexpected answers far from the respondent's measure level (person logit). Among other considerations are to reword these items or exclude them from the measurement.

Our results also showed the adequacy of the rating scale categories, with enough separation between them, low SE of thresholds, and ordered delta values on the Rasch–Andrich threshold measure. Therefore, all the categories of response were likely to be chosen (Linacre, 2006), supporting the use of the 4-point rating scale in this instrument.

Finally, the differential-item functioning analysis showed differences in the functioning of the items regarding the age groups of children. Some variability was expected due to the age of the group variable. In order to interpret these results, it is necessary to consider the age of the item (i.e., the age it would be expected a child to participate in the routine in the way described by the item) and the age groups considered and compared. Eight items showed statistically significant differences at *p* < 0.0007 (i.e., 1.1, 1.4, 1.5, 2.5, 4.2, 4.3, i5.1, i5.5). For four out of these eight, the direction of the differences in item difficulty followed the expected pattern by item and the age groups been compared. These items were aged at 36 to 48 months (i.e., i1.5) or 40–60 months (i.e., i4.2, i4.3, and 5.5). The item measure for younger children was expected to be higher and lower for older children, given that children in the 3-year old group are just learning these abilities to participate in the routines. However, items 1.1, 1.4, 2.5, and 5.1 showed differences between age groups that did not follow the expected pattern of functioning given the items age and the age of the comparison groups. It is concluded that either the age or the wording of these items must be revised to avoid bias. Moreover, for interpreting these differences among functioning of items, it is also relevant to consider the routine in which the item is included. The skills represented by the items may be more encouraged and supported at younger ages, more than at older ages, therefore children used these skills to participate in the routine more often than older children, who are still learning them and practicing them but do not receive reminders or support as younger children do. This may help to explain, why some items were less difficult to be performed with higher frequency for younger children than older children. Following the ICF framework for interpreting functioning (World Health Organization, 2001), it is understood that contextual factors (i.e., demands of the routine) and personal factor (i.e., child interests) could have an impact on the child's participation. Therefore, the wording of the items should be revised so that teachers can evaluate the degree of functioning of the child at a specific independence level. This is in order to reduce bias due to children's ability to complete the items either with some support or none from the teacher. As children may be participating in some routines with a lot of support from the teacher, therefore difficulty of the item is lower for younger children than for older children, who are no receiving as much support by the teacher. Future empirical efforts could explore this relation, going beyond the assessment of participation, and determining the effect of environmental and contextual factors on child participation in early childhood classroom routines. Thus, the understanding of these items and its rating will be easier, and bias will be avoided.

## What This Paper Adds?

Our findings support the adequacy of the 3M scale for capturing children's low and average levels of functioning in preschool classroom routines. We did, however, identify some weaknesses. More difficult items need to be included in the scale to match different levels of functioning of children and to avoid ceiling-effects, especially for those children with higher ability levels, and some items need to be reworded to avoid bias due to lack of specificity on the level of independence of the child while performing the skill described by the item. Table 6 presents the original 3M items and the new proposed items after considering the results of this study. Furthermore, this table shows the ICF codes, and its definition, associated to each scale item.

**Table 6:** Original items, new items, and ICF codes.

**Routine**	**Item**	**Original**	**New**	**ICF code**	
Eating	1.1	Uses fork and spoon to stab and scoop food^a^	Grabs his/her lunch and eats it independently	d550	Eating
	1.2	Drinks from cup without spilling content	–	d560	Drinking
	1.3	Initiates communication with peers^a^	Initiates conversations with peers (i.e., tries to get a peer's attention by calling their name or starting a conversation)	d350	Conversation
	1.4	Clears table after eating (throws away trash/puts away food containers) without been prompted^a^	Throws away trash/puts away food containers after eating without been prompted.	d640	Doing Housework (collecting and disposing garbage)
	1.5	Uses words, signs, and/or gestures to express needs to the teacher and peers	Uses words, signs, and/or gestures to express needs to the teacher and peers	d330	Speaking
Free play	2.1	Engages in pretend play by acting out scenarios	Engages in pretend play by acting out scenarios	d131	Learning through actions with objects
	2.2	Independently chooses and obtains accessible materials	Independently chooses and obtains accessible materials	d177	Making decisions
	2.3	Cooperates with peers while playing (e.g., negotiates roles)	Cooperates with peers while playing without been prompted (e.g., negotiates roles)	d750	Informal Social Relationships
	2.4	Talks to peers using understandable language	Talks to peers using understandable language	d330	Speaking
	2.5	Shows empathy toward the feelings of other people^a^	Shows empathy toward the feelings of other people without been prompted	d720	Complex interpersonal interactions
	2.6	–	Participates in play by moving (e.g., runs, rides a tricycle, goes down stairs, moves using mobility equipment such as walkers or wheel-chairs)	d435	Moving objects with lower extremities
	2.6	–	Participates in play by moving (e.g., runs, rides a tricycle, goes down stairs, moves using mobility equipment such as walkers or wheel-chairs)	d445	Hand and arm use
	2.7	–	Participates in games that required coordination and balance (e.g., swings, rides a bicycle, balances in one foot, goes up the slide stares, throws, and catches a ball)	d435	Moving objects with lower extremities and Moving around
	2.7	–	Participates in games that required coordination and balance (e.g., swings, rides a bicycle, balances in one foot, goes up the slide stares, throws, and catches a ball)	d455	Hand and arm use
Toileting	3.1	Urinates in potty with no accidents^a^	Goes into the bathroom independently without accidents (e.g., goes to the toilet by him-/herself, wipes by him-/herself, pulls the chain)	d530	Toileting
	3.2	Washes his/her hands after using the toilet	–	d510	Washing oneself
	3.3	Uses zipper, snap, or buttons	Uses zipper, snap, and buttons	d540	Dressing
	3.4	Dresses and undresses without assistance	Dresses and undresses without assistance	d540	Dressing
	3.5	Goes into bathroom independently or asks for permission by using words or signs^a^	Asks permission to go into the bathroom using words, signs, or gestures	d330	Speaking
	3.5	Goes into bathroom independently or asks for permission by using words or signs^a^	Asks permission to go into the bathroom using words, signs, or gestures	d340	Producing messages in formal sign language
Art	4.1	Respond to 3-step instructions from the teacher	Respond to 3-step instructions from the teacher	d310	Communicating with-receiving-spoken messages
	4.2	Makes representational art (e.g., draws, paints, or builds things to look like real objects)	Makes representational art (e.g., draws, paints, or builds things to look like real objects)	d335	Producing non-verbal messages
	4.3	Uses scissors independently	Uses scissors independently	d445	Hand and arm use
	4.4	Talks about his or her art product in full sentences	Talks about his or her art product in full sentences	d330	Speaking
	4.5	Waits for his or her turn to use materials without getting upset	Waits for his or her turn to use materials without getting upset	d720	Complex interpersonal interactions
Teacher-led activities	5.1	Attends to teacher when he or she is talking to the group for periods of time longer than 5 min^a^	Attends to teacher when he or she is talking to the group from the beginning to the end of the teacher's intervention.	d160	Focusing attention
	5.2	Participates in group activities that involve communication, by using full sentences	Participates in group activities that involve communication, by using full sentences	d350	Conversation
	5.3	Jumps by lifting both feet from the ground^a^	Participates in singing or games by jumping and lifting both feet from the ground^a^	d332	Singing
				d445	Moving around
	5.4	Imitates teacher's gestures and movements while singing	Imitates teacher's gestures and movements while singing	d130	Copying
	5.5	Follows rules and teacher's requests^a^	Is compliant with the classroom rules and teacher's requests	d310	Communicating with-receiving-spoken messages
	5.6	–	Participates in conversations by using the past- and future-tense	d350	Conversation

a *Represent items that have been reworded or added*.

In order to develop a functional profile for the child based on the 3M scale items, the rating scale could be used to determine the degree of difficulty the child encounters to perform or participate in the routine. Teachers are encouraged to use these qualifiers to understand the level of difficulty for participation in routines when accounting specific items. For example, if a teachers rates a child's performance on item 3.4 “Dresses and undresses without assistance” as a “2,” this score would indicate a high difficulty for participating in toileting. Then, teachers could evaluate if (1) more supports are needed because of lack of capacity of the child, (2) the demands of the routines exceed the child's capacities therefore representing a barrier for his or her participation and should be modified, or (3) it is necessary to teach the child abilities for promoting his or her performance in this routine (Morales Murillo, 2018). Table 7 shows the cross-walk among the scale categories of response and the ICF performance qualifiers.

**Table 7:** Cross-walk between the 3M scale response categories and the ICF performance qualifiers.

**3M scale**		**ICF performance qualifier**		
**Response category**	**Score**	**Qualifier**	**Definition**	**%**
Not yet	1	dxxx.4	Complete difficulty (total,…)	96–100
Rarely	2	dxxx.3	Severe difficulty (high, extreme,…)	50–95
Often	3	dxxx.2 or dxxx.1	Moderate (medium, fair,…) or Mild difficulty (slight, low,…)	Moderate (25–49) or Mild (5–24)
Almost all the time	4	dxxx.0	No difficulty (none, absent, negligible,…)	0–4

If a child is rated with low scores in the 3M scale items, meaning the child is encountering activity limitations or participation restrictions, this could indicate a poor fit between (a) the child's capabilities and personal interests and (b) the demands of the routine (environmental factors), which may have an impact in the child's performance. Then, teachers can use this information to determine if the low performance of the child is the result of low capacity or a mismatch between environmental barriers and the child's personal factors or capabilities. Therefore, supports can be incorporated into the routines to facilitate the child's meaningful participation. Further assessment may be need to determine a full functional profile of the child. For this purpose, the ClaMEISR (McWilliam, 2014) represents an useful tool.

Finally, because multidimensionality was not supported by the results of the first run rasch, and after deleting items undimensionallity of the scale was supported, authors recommend the used of the whole score as an indicator of children's functioning in the preschool classroom. In addition, individual items could be used to determine the degree of difficulty for performing such item in a given routine. Reflection upon the child‘s performance and the analysis of barriers and facilitators that may be hindering or enhancing the performance of the child in a given routine is encouraged.

## Limitations and Future Lines

Future research should study the social and the construct validity, using other validated tools for measuring child functioning such as the Matrix for Assessment of Activities and Participation developed by Castro and Pinto (2015). It could include the 3M scale with the changes suggested in this study.

## Conclusions

Our data suggest the 3M is useful for evaluating children's functioning in preschool classroom routines. Because the 3M includes assessment contexts (i.e., routines), it provides a link between the child's activities and participation (i.e., engagement, independence and social relationships) and the context factors, supporting the reflection of teachers about children's meaningful participation in classroom routines. Teachers can use this tool to identify the classroom routines where children are struggling to meaningfully participate, and from there, analyses the barriers and facilitators that could be diminished or spurred the activity or participation of the child, so that barriers are eliminated and facilitators are provided to ensure child's functioning in classroom routines.

## Data Availability Statement

The datasets presented in this article are not readily available because data contains child information that cannot be shared. If needed, authors could share data files without participants information (only scale-items raw-scores). Requests to access the datasets should be directed to Catalina Patricia Morales-Murillo, catalina.morales@unir.net.

## Ethics Statement

The studies involving human participants were reviewed and approved by the University of Tennessee at Chattanooga Institutional Review Board (IRB # 14-095). Written informed consent to participate in this study was provided by the participants' legal guardian/next of kin.

## Author Contributions

All authors listed have made a substantial, direct and intellectual contribution to the work, and approved it for publication.

## Conflict of Interest

The authors declare that the research was conducted in the absence of any commercial or financial relationships that could be construed as a potential conflict of interest.
